# SuperSour: A New Strategy for Breeding Superior Citrus Rootstocks

**DOI:** 10.3389/fpls.2021.741009

**Published:** 2021-11-04

**Authors:** Kim D. Bowman, Greg McCollum, Ute Albrecht

**Affiliations:** ^1^U.S. Horticultural Research Laboratory, Agricultural Research Service, United States Department of Agriculture, Ft. Pierce, FL, United States; ^2^Southwest Florida Research and Education Center, Horticultural Sciences Department, Institute of Food and Agricultural Sciences, University of Florida, Immokalee, FL, United States

**Keywords:** fruit yield, genetic mapping, citrus rootstock, breeding strategy, huanglongbing disease

## Abstract

Citrus crops have a long history of cultivation as grafted trees on selected rootstock cultivars, but all current rootstocks have significant limitations and traditional methods of rootstock breeding take at least 2–3 decades to develop and field test new rootstocks. Citrus production in the United States, and other parts of the world, is impaired by a wide range of biotic and abiotic problems, with especially severe damage caused by the disease huanglongbing (HLB) associated with *Candidatus* Liberibacter asiaticus. All major commercial citrus scion cultivars are damaged by HLB, but tree tolerance is significantly improved by some rootstocks. To overcome these challenges, the USDA citrus breeding program has implemented a multi-pronged strategy for rootstock breeding that expands the diversity of germplasm utilized in rootstock breeding, significantly increases the number of new hybrids evaluated concurrently, and greatly reduces the time from cross to potential cultivar release. We describe the key components and methodologies of this new strategy, termed “SuperSour,” along with reference to the historical favorite rootstock sour orange (*Citrus aurantium*), and previous methods employed in citrus rootstock breeding. Rootstock propagation by cuttings and tissue culture is one key to the new strategy, and by avoiding the need for nucellar seeds, eliminates the 6- to 15-year delay in testing while waiting for new hybrids to fruit. In addition, avoiding selection of parents and progeny based on nucellar polyembryony vastly expands the potential genepool for use in rootstock improvement. Fifteen new field trials with more than 350 new hybrid rootstocks have been established under the SuperSour strategy in the last 8 years. Detailed multi-year performance data from the trials will be used to identify superior rootstocks for commercial release, and to map important traits and develop molecular markers for the next generation of rootstock development. Results from two of these multi-year replicated field trials with sweet orange scion are presented to illustrate performance of 97 new hybrid rootstocks relative to four commercial rootstocks. Through the first 7 years in the field with endemic HLB, many of the new SuperSour hybrid rootstocks exhibit greatly superior fruit yield, yield efficiency, canopy health, and fruit quality, as compared with the standard rootstocks included in the trials.

## Introduction

The genus *Citrus* encompasses a broad range of fruit crops of worldwide importance, including sweet orange (*Citrus sinensis* [L.] Osbeck), mandarin (*C. reticulata* Blanco), grapefruit (*C. paradisi* Macf.), and lemon (*C. limon* [L.] Burm. f.), all of which are usually grown in modern citriculture as grafted trees on selected specialized rootstock cultivars. Although most selection of citrus in early history focused on fruit and above-ground tree characteristics, many natural citrus selections with useful rootstock characteristics were identified before the beginning of modern directed citrus breeding (Bitters et al., 1969). These natural selections provided the major rootstocks employed during the early use of grafted citrus trees in the 19th century. Directed citrus breeding for improved scions began in the late 19th century (Cooper et al., 1962), and related interest in breeding for improved rootstocks began shortly afterward. As a result of selection among natural variants and directed breeding efforts, there are now hundreds of different rootstock clones with some commercial use in the many countries involved in citrus production. In Florida, more than 40 different rootstocks are used for propagation in commercial citrus nurseries each year (Rosson, 2020), as growers attempt to find the best rootstock for each situation. Unfortunately, all currently used rootstocks have known or indeterminate faults (Bowman and Joubert, 2020), which limit their survival, tolerance of disease, adaptability, or positive influence on fruit productivity or quality.

In particular, threats from a range of common diseases and pests have continued to reduce tree health and cause major losses in terms of tree survival and productivity in most production regions. The disease huanglongbing (Bove, 2006), associated with *Candidatus* Liberibacter asiaticus (CLas) and other *Ca.* Liberibacter species, is widespread in the Eastern Hemisphere and has spread to most of the citrus production area in the Western Hemisphere over the last 15 years. In Florida and Brazil, the disease is causing devastating declines in productivity (Alvarez et al., 2016; Bassanezi et al., 2020; Li et al., 2020) and fruit quality (Dala-Paula et al., 2019). Perhaps of the greatest commercial significance, all existing sweet orange and grapefruit cultivars and many mandarins are highly susceptible and severely affected by huanglongbing (HLB) disease. While specialized intensive management practices can somewhat slow disease spread and lessen the disease effects (Stansly et al., 2014), these practices have limited effectiveness and incur significant costs that often eliminate the profitability of growing the citrus crop. In addition, many of the management practices required to effectively moderate disease spread and limit decline in production involve intensive use of pesticides and nutritional treatments that threaten the environment. As a partial solution, good tolerance to CLas infection has been demonstrated in citrus germplasm, and some existing rootstocks such as US-942, have demonstrated field tolerance to HLB that can significantly improve health and productivity of sweet orange trees in an HLB-endemic environment (Bowman and McCollum, 2015; Bowman et al., 2016a, b). However, even with the use of the best of the currently available HLB-tolerant rootstocks, sweet orange and grapefruit trees suffer a significantly reduced tree health, and loss in fruit production and fruit quality, as compared to non-infected trees. Improved citrus rootstocks are urgently needed to provide better tolerance or resistance to diseases such as HLB, as well as improved influence on fruit production and fruit quality of the scion. While traditional citrus breeding efforts by USDA and others have been historically successful in producing new rootstocks of commercial value (Bowman and Joubert, 2020), the pace of these efforts has been very slow, taking several decades from cross to commercial release. In addition, previous conventional citrus rootstock breeding by USDA and several other programs focused on relatively limited portions of the citrus genepool because of an obligatory apomictic seed reproduction that has characterized essentially all citrus rootstocks in commercial use. In this article, we describe a new multi-pronged strategy for citrus rootstock breeding in the USDA program, which includes greater genetic diversity and expanded concurrent early-stage testing of a large number of new hybrids. The strategy significantly reduces the time from cross to release of a new hybrid rootstock by eliminating apomictic seed reproduction as a prerequisite for field-testing and as a requirement for new rootstocks. Although uniform seed propagation is convenient and economical for the citrus nursery, the historical focus on nucellar polyembryony as a primary rootstock trait has become a critical limitation in traditional citrus rootstock breeding. The expanded concurrent and replicated testing of hundreds of new hybrid rootstocks also is providing extensive genotype-performance information that will be used to map important genetic traits and develop molecular markers to power the next generation of citrus rootstock breeding.

## History of Citrus Rootstocks and Breeding

Early cultivation of citrus crops undoubtedly relied on seedling trees chosen for their favorable fruit quality traits. Some citrus species and selections have a reproductive trait known as nucellar polyembryony (Frost and Soost, 1968; Koltunow et al., 1995; Kepiro and Roose, 2007), which causes most seedlings to be derived by apomixis from maternal tissue and genetically identical to the seed source tree. In citrus, the apomictic production of seedlings for many species and selections made rapid and uniform seed propagation of many fruiting types quite easy, and probably had an important role in early selection of those types that were easy to propagate by seed. The existence of nucellar polyembryony, and resulting easy uniform propagation by seed, in sweet orange, sour orange (*Citrus aurantium* L.), and lemon was probably a critical reason for the widespread distribution of these types in early history, as seedlings grown for their fruit.

There is mention of grafting citrus in historical accounts in Chinese, Greek, and Roman literature from 1000 to 2000 years ago (Bitters, 1986), but it appears that this was usually as a curiosity for citrus in botanical collections, arboretums, and orangeries (Webber, 1967), rather than a practice for widespread fruit production. Undoubtedly, the ease of propagating many citrus fruiting varieties with good fidelity through nucellar seedlings reduced the motivation for grafting and rootstock use in these crops. Historical accounts suggest the first significant use of grafted trees for large-scale citrus production began about the mid- to late 1800s in the United States, and was driven primarily by *Phytophthora* disease problems on seedling trees in Florida (Castle, 2010), and the desire to vegetatively propagate specific clones of seedless sweet orange (such as Washington Navel orange) in California (Bitters, 1986). Initially, the use of grafting in citrus commercial production appears to have been as topworking of existing seedling trees growing in the field, rather than production in a tree nursery. The topworking of wild sour orange groves with sweet orange in Florida began as early as 1830, but was very successful and became widespread by the late 1800s (Webber, 1967). Through the end of the 19^th^ century, all citrus varieties in existence were natural species or the result of natural hybridizations, and any efforts at using citrus rootstocks relied upon those naturally occurring varieties.

The rootstock with greatest fame and notoriety may be sour orange, or the genetic clone that has long been classified as *Citrus aurantium*. Sour orange was originally selected for cultivation because of its fragrance and fruit that was valued for flavoring fish and meat (Tolkowsky, 1938), rather than as a fresh fruit or a rootstock. Over time, interest in sour orange as a fruiting variety declined, but it came to be recognized as possessing many excellent rootstock traits, including uniform seed propagation, broad soil adaptability, and good influence on fruit cropping and fruit quality of a grafted scion. Sour orange was probably the most often used rootstock during the early era of grafted citrus trees in most citrus regions. In Florida, established groves of sour orange seedlings were topworked with sweet orange during the mid-1800s with great success, and contributed to the rapid expansion of the citrus industry in Florida (Webber, 1967). However, sour orange also has a flaw in its sensitivity to citrus tristeza virus (CTV) when used as a rootstock with most scions, and there are numerous examples of disastrous citrus industry losses because of CTV-induced quick decline in trees on this rootstock. Historically, major tree losses from CTV infection of citrus trees on sour orange rootstock have been reported in Argentina, Brazil, Ghana, and California (Bar-Joseph et al., 1989). Despite this risk, sour orange still has some popularity in many citrus growing areas and is regarded by many as one of the best citrus rootstocks (except for the Achilles heel of CTV sensitivity) because of its good adaptability to unfavorable soil conditions and good influence on fruit quality. One of the citrus production areas where sour orange is still regarded very highly is the Indian River Region of Florida, where it is especially considered as an outstanding rootstock for grapefruit (Castle et al., 2011). Although sour orange is given species status in botanical classification, it is now identified from several different lines of evidence, including nuclear (Wu et al., 2014) and chloroplast (Carbonell-Caballero et al., 2015) sequencing studies, to be derived from a single natural hybridization of pummelo (*Citrus maxima* [L.] Osbeck) as the seed parent, and mandarin (*Citrus reticulata*) as the pollen parent. That natural hybridization probably occurred about 3,000 years ago (Carbonell-Caballero et al., 2015). Over the last 30 centuries, point mutations have created a multitude of sour orange variants with similar fruit and horticultural traits (Bowman and Garnsey, 2001), and the many small variations on this interspecific hybrid have been spread widely through genetically identical apomictic seedlings produced by nucellar polyembryony.

The clearest evidence of a transition to a more scientific approach to breeding new citrus varieties was in the establishment of the USDA Subtropical Laboratory at Eustis, Florida, in 1892, where H. J. Webber and W. T. Swingle began research on citrus diseases and breeding. Over the following 30 years, similar efforts to begin scientific research directed at solving citrus problems, in part through breeding, began at University of California, University of Florida, and in Europe, South Africa, and Japan (Webber, 1967). The earliest focus of breeding in the USDA program in Florida was to obtain fruiting varieties with greater cold hardiness, a response to the disastrous freeze in the winter of 1894/1895. This effort, which focused most heavily on crossing the cold hardy citrus relative trifoliate orange (*Poncirus trifoliata* [L.] Raf.) with sweet orange and grapefruit, was not successful in producing cold hardy scion varieties because of very acrid fruit flavors found in first- and second-generation hybrids of citrus with trifoliate orange. However, out of this effort a series of trifoliate orange hybrids were produced which eventually became recognized as having good rootstock traits. Two of these trifoliate hybrids, Carrizo citrange (sweet orange × trifoliate orange) and Swingle citrumelo (grapefruit × trifoliate orange) were eventually released by USDA as rootstocks, in 1934 and 1974 (Hutchison, 1974; Wutscher, 1979), respectively, and have become major rootstocks with commercial use in all regions of the world (Bowman and Joubert, 2020). The USDA citrus rootstock breeding program was revived beginning in 1992, and the program released several other trifoliate hybrids that became important in Florida production during 2001–2020 (Bowman et al., 2016a). Testing of candidate hybrid rootstocks under the USDA breeding program has included focused evaluation of selected traits of horticultural importance, including nursery traits (Bowman et al., 1995, 1997, 2016a; Barcelos-Bisi et al., 2020) and tolerance to diseases and pests (Bowman and Garnsey, 2001; Bowman et al., 2001, 2002, 2003; Albrecht and Bowman, 2004, 2007, 2011, 2012, 2019; Graham et al., 2007; Xiang et al., 2010; Bowman and Albrecht, 2015, 2020), but focus of evaluation and selection was on field performance under typical commercial production conditions. Similar rootstock breeding efforts to cross trifoliate orange with sweet orange, grapefruit, and mandarin were also conducted at University of Florida (Castle, 1987; Castle et al., 1988) and University of California (Bitters et al., 1964; Cameron and Soost, 1986), as well as by researchers in Europe (Forner et al., 2003), South Africa (von Broembsen, 1985; Lee et al., 2009), and Australia (Long et al., 1977; Freeman et al., 1986). Other than work with the USDA, recent rootstock breeding efforts are best documented for programs at University of Florida (United States), Valencian Institute of Agrarian Research (IVIA, Spain), Council for Agricultural Research and Economics (CREA, Italy), and Empresa Brasileira de Pesquisa Agropecuária (EMBRAPA, Brazil).

At University of Florida, active work to breed new citrus rootstocks has been underway by a team at Lake Alfred for more than 35 years. Much of the early work by the team made use of somatic hybridization, and then subsequent crosses among allotetraploid products of that prior work (Grosser et al., 2004; Grosser and Gmitter, 2011). Sexual hybridization among diploid parents was also used in the UF program to develop new rootstock hybrids. During the evolution of this program, the UF team began to use “the gauntlet” method for screening new rootstock hybrids, which involves the screening of individual hybrid seedlings sequentially by high pH, *Phytophthora* infestation, and HLB exposure to identify those most hardy to survive these stresses, followed by a field trial with the survivors (Grosser et al., 2016, 2020). Nine new UF citrus rootstocks were released in 2013 (UFR-1, UFR-2, UFR-3, UFR-4, UFR-5, UFR-6, UFR-15, UFR-16, and UFR-17)^1^. The most popular of those is UFR-4, which was used for 49,057 Florida propagations in 2019–20 (Rosson, 2020).

At IVIA (Valencia, Spain), conventional sexual hybridization to develop superior new citrus rootstocks has been underway since the 1970s (Martínez-Cuenca et al., 2016). During the later stages, the IVIA program emphasized and documented information on screening and selection of hybrids for resistance to salinity (Forner-Giner et al., 2009), iron deficiency (Llosa et al., 2009), and water stress (Rodriguez-Gamir et al., 2010). Validating tolerance to citrus nematode *Tylenchulus semipenetrans* (Verdejo-Lucas et al., 1997) and *Phytophthora* species found in Spain has also been an important part of rootstock selection, along with highly uniform seedling propagation (Forner et al., 2003). The rootstocks released from the program include F-A 5, F-A 13, F-A 418, and F-A 517, with the latter two being considered dwarfing (Forner-Giner et al., 2014). Of these, F-A 5 has had the most commercial interest, with considerable plantings using this rootstock in Spain and nearby citrus production regions.

In the 1960s, the CRA-Research Center for Citriculture and Mediterranean Crops (CRA-ACM) initiated a conventional citrus rootstock breeding program in Sicily, Italy. Selections were made from among progeny from crosses for tolerance to “mal secco” and *Phytophthora*, and for nucellar embryony and vigor, followed by field trials (Russo and Reforgiato Recupero, 1984; Reforgiato Recupero and Russo, 1992). Out of this work, CREA (Acireale, Italy) released three hybrids of *C. latipes* (Tan.) × *P. trifoliata* (F5P12, F6P12, F6P13) that demonstrated high yield in field trials with several different scions (Reforgiato Recupero et al., 2009). In a recent rootstock trial with triploid Mandared scion in Siracusa (Italy), this team found that some of the rootstocks from California (including C35) were superior to the CREA hybrids (Caruso et al., 2020).

Citrus production in Brazil is primarily on Rangpur rootstock, which is well suited to rainfed cultivation and the primary crop of that region, juice oranges. But there is still great interest in diversifying rootstock use and finding better rootstocks for specialty scions, different production areas in the region, and to cope with HLB and other specific problems, motivating active breeding and evaluation under EMBRAPA and at The Sylvio Moreira Citriculture Center (Institute Agronomic of Campinas, Cordeirópolis), as well as work under Fundecitrus, IDR-Paraná (Rural Development Institute of Paraná), and University of São Paulo (ESALQ – USP). Hybrid and clonal rootstocks from Brazil and other countries are evaluated for nursery performance (Marques et al., 2021), drought (Santana-Vieira et al., 2016), and tree size control (Costa et al., 2021). Numerous field trials have been used to compare performance of the many rootstocks, with superior rootstocks identified for the different production situations (Cantuarias-Avilés et al., 2010; Girardi et al., 2017; Carvalho et al., 2019; Costa et al., 2020b; de Carvalho et al., 2021). Focused studies were conducted to map quantitative trait loci (QTL) in citrandarins associated with host response to infection by CLas (Soratto et al., 2020) and *Phytophthora parasitica* (Lima et al., 2018), and to identify mechanisms of tolerance to HLB (Curtolo et al., 2020). A series of non-parametric indices were evaluated for selection of superior hybrid rootstocks, and a ranking index was identified as the most useful for selecting productive and drought-tolerant rootstocks (Costa et al., 2020a).

To date, the only hybrid citrus rootstocks from breeding programs that have gained major commercial importance (used for more than 5% of nursery production over several years in at least one important crop region; as reported in Bowman and Joubert, 2020) besides Carrizo and Swingle (Table 1), are Benton, C35, and Kuharske citranges (*C. sinensis* × *P. trifoliata*), X639, F-A 5, US-812, US-897, and US-942 citrandarins (*C. reticulata* × *P. trifoliata*), US-802 (*C. maxima* × *P. trifoliata*), and MxT [(*C. paradisi* × *C. reticulata)* × *P. trifoliata*)]. The other rootstocks of major commercial importance are naturally occurring species or selections, including Cleopatra and Sunki mandarins, clones of trifoliate orange (*Poncirus trifoliata)*, Rangpur, Rough lemon, Volkamer, and Ziyang Xiangcheng, and these all exhibit nucellar polyembryony. All the commercially important products from breeding programs are F1 hybrids of trifoliate orange with citrus, and all possess nucellar polyembryony, a trait that is obtained in a large portion of progeny from crosses of trifoliate orange with many citrus species. Efforts have been made to expand the range of parentage included in new rootstock hybrids, such as the *C. latipes* × *P. trifoliata* hybrid rootstocks from CREA-Italy (Reforgiato Recupero et al., 2009), but none of these have yet gained commercial acceptance.

**TABLE 1:** Major citrus rootstocks of the world, with regions of primary use, parentage (for those from known crosses), date commercial use began, and uniformity of seed propagation.

Rootstock	Current regions of primary commercial use^a^	Parentage	Beginning of use as rootstock	Uniform seed propagation
Benton	SU	*Citrus sinensis* × *Poncirus trifoliata*	1990	Yes
Carrizo	NCSEFMAU	*C. sinensis* × *P. trifoliata*	1934	Yes
Cleopatra	NCSEMU	*C. reticulata*	Before 1900	Yes
C35	NCSEFMU	*C. sinensis* × *P. trifoliata*	1986	Yes
F-A 5	E	*C. reticulata* × *P. trifoliata*	2003	Yes
Kuharske	N	*C. sinensis* × *P. trifoliata*	Before 2000	Yes
Macrophylla	NEMU	*C. macrophylla*	Before 1960	Yes
MXT	F	*(C. paradisi* × *C. reticulata)* × *P. trifoliata*	1992	Yes
Trifoliate orange	NCSEMAU	*P. trifoliata*	Before 1900	Yes
Rangpur	CSA	*C. limonia*	Before 1900	Yes
Rough lemon	NCFA	*C. jambhiri*	Before 1900	Yes
Sour orange	NCSEMA	*C. aurantium*	Before 1850	Yes
Sunki	S	*C. sunki*	Before 1900	Yes
Swingle	NCSEFAU	*C. paradisi* × *P. trifoliata*	1974	Yes
US-802	N	*C. maxima* × *P. trifoliata*	2007	Yes
US-812	NM	*C. reticulata* × *P. trifoliata*	2001	Yes
US-897	N	*C. reticulata* × *P. trifoliata*	2007	Yes
US-942	N	*C. reticulata* × *P. trifoliata*	2010	Yes
Volkamer	NCSEFMAU	*C. volkameriana*	Before 1970	Yes
X639	NCF	*C. reticulata* × *P. trifoliata*	2000	Yes
Ziyang Xiangcheng	A	*C. junos*	2010	Unknown

*^a^N, North America; C, Central America and Caribbean; S, South America; E, Europe; F, Southern Africa; M, North Africa and Middle East; A, Asia; U, Australia.*

Over the last 35 years, research efforts to develop improved citrus rootstocks have expanded at many institutions to include new methodologies (Salonia et al., 2020), including ploidy manipulations, somatic hybridization, genetic transformation, and genome editing. Selections of rootstock clones with a doubling of chromosomes, or autotetraploids, can often be visually identified among groups of seedlings and have received interest for more than 80 years (Lapin, 1937; Russo and Torrisi, 1951; Barrett and Hutchison, 1978; Bowman et al., 1991; Ollitrault et al., 2020). There is evidence that some autotetraploids have greater stress tolerance than their diploid twin (Allario et al., 2013; Oliveira et al., 2017; Oustric et al., 2017), but none of these tetraploid selections have become important rootstocks in commercial use. Considerable effort has been invested and progress made in somatic hybridization directed at rootstock improvement at University of Florida (Grosser et al., 1998), and in France (Dambier et al., 2011) and Spain (Ruiz et al., 2018), after the technique was first demonstrated in Japan (Ohgawara et al., 1985). Most initial work with somatic hybrids was to create allotetraploid hybrids among existing rootstocks (Dambier et al., 2011) and with other species (Grosser et al., 1996). Subsequent work included the creation of cybrids (Moreira et al., 2000) and has followed with sexual hybridization among the tetraploids (Grosser et al., 2015). New rootstocks that are the product of somatic hybridization have been released in Florida (Kunwar et al., 2021), although so far none of those from Florida or other programs have become of major commercial importance.

*Agrobacterium*-mediated genetic transformation has been applied toward improvement of citrus rootstocks for more than 25 years (Cervera et al., 1998; Benyon et al., 2013), but significant challenges remain to the development and commercial acceptance of the GMO products (Song et al., 2019). Current public attitudes toward the products of *Agrobacterium*-mediated genetic modification seem to limit applications in fruit crops (Lucht, 2015); but the potential to use GMO rootstocks to improve the health and productivity of trees with non-GMO scions has been proposed as one way to avoid this problem (Haroldsen et al., 2012; Song et al., 2015). Methods to excise the selectable markers in transgenic citrus plants have been demonstrated (Zou et al., 2013) and proposed as another way to alleviate GMO restrictions and reduce public concerns. Introduction of early flowering genes by genetic transformation to accelerate the breeding cycle of fruit tree crops, followed by backcrossing to remove those genes before commercialization of the new cultivars, have also been proposed as a method to use *Agrobacterium*-mediated transformation for genetic improvement and circumvent GMO-related concerns (Petri et al., 2018). Induction of early flowering through transient expression by a viral vector has been demonstrated in citrus (Velazquez et al., 2016), and is yet another approach to using an early-flowering transgene to accelerate citrus breeding, while potentially avoiding a GMO-cultivar product.

Genome editing avoids some of the regulatory limitations of genetic transformation and has been demonstrated in citrus (Jia and Wang, 2014). Some progress has also been made in efforts to use genome editing to improve disease resistance (Peng et al., 2017; Jia et al., 2019; Wang et al., 2019). Although numerous groups have made very large investments of resources into *Agrobacterium*-mediated transformation and genome editing of citrus (Poles et al., 2020), as of now, no citrus rootstocks derived from GMO technology or genome editing have become commercially available. While new technologies offer abundant options for continuing work, none has yet proven successful in creating rootstocks of commercial importance, and it seems likely that conventional breeding will continue to have the primary role in the development of the next generation of citrus rootstocks in the coming decade.

## Need for Better Rootstocks and a New Strategy

Citrus rootstock breeding programs are expensive and require very long-term investment that is generally not suited to the objectives of private industry, leaving such research to government or university sponsored programs. Despite considerable effort toward developing and testing new citrus rootstocks at numerous institutions and citrus growing regions over the past 100 years, inadequate rootstocks are still used for a large portion of world citrus production. Rootstock trials in different regions demonstrate, repeatedly, that trees on some particular rootstocks show better tolerance to disease and stress, and yield more and better quality fruit than other rootstocks (Louzada et al., 2008; Rodriguez-Gamir et al., 2010; Bowman et al., 2016a; Cruz et al., 2019; Kunwar et al., 2021; Singerman et al., 2021). Within a regional rootstock trial, often the best performing rootstocks are not those that are in most common commercial use. One impediment to adoption of better rootstocks in commercial plantings is the long life of a citrus tree and the expectation of multiple-decade production from a tree. It is often considered uneconomical to replace an existing mature bearing tree, even if the replacement tree might eventually become more productive. However, of greatest importance in limiting the development and commercial adoption of superior new rootstocks are: (1) the limited genetic diversity of germplasm used in creating new hybrids and compared in individual field trials, (2) the limited number of rootstocks in trials and locations of trial comparisons among rootstocks, so that growers do not have enough information to be sure about the best choice in broader regions, (3) the slow pace of new rootstocks being available from commercial nurseries, when obligate apomictic seed propagation is required, and (4) the inability to select the best genetic combinations at an early stage of rootstock development, resulting in large resource utilization in testing many unfavorable candidate rootstocks.

To address these problems with new rootstock development and the adoption of superior rootstocks by industry, the USDA citrus breeding program in Fort Pierce, FL (United States) began a new strategy for rootstock development in 2006. The new strategy was termed “SuperSour” because the target was to create a superior new hybrid rootstock that possesses the best rootstock traits of sour orange (*C. aurantium*) along with superior tolerance to HLB and other biotic and abiotic problems and superior production of high-quality fruit, while avoiding sensitivity to CTV-related tree decline. The strategy includes *C. maxima* and *C. reticulata* (the two parental species of sour orange) in most hybrids, along with *P. trifoliata* and other species to introduce additional positive rootstock traits. The primary features of this new SuperSour strategy are: (1) the expanded gene pool used in new rootstock hybrids, including germplasm that was not previously used (such as *C. maxima*) because it does not transmit nucellar polyembryony to a high portion of progeny, (2) accelerated field testing of new hybrids by propagating the hybrids with stem cuttings, rather than waiting for fruiting and seeds, (3) expanded concurrent stage 1 field testing of new hybrids in multiple production sites and multiple planting dates, that can be cross-compared by using the same standard rootstocks in each trial, (4) release of new rootstocks based on superior performance in multiple field trials, and without a requirement for propagation by nucellar polyembryony, and (5) assembly of standardized multi-year performance data on hundreds of hybrid rootstocks from multiple trials, which can be used to genetically map important rootstock traits and develop molecular markers to streamline future rootstock breeding.

The new strategy includes evaluation and testing for nursery traits, and tolerance or resistance to abiotic and biotic factors. But this testing is done concurrently with field trials of the candidate hybrid rootstocks, rather than as a prerequisite for inclusion in field trials. While nursery traits like nucellar polyembryony and vigorous nursery growth are valuable, they do not directly affect the field performance of the rootstock. Tolerance or resistance to abiotic and biotic factors are valuable for field performance, but survival, health, and fruit productivity in the field is the combination of numerous interrelated traits which are best evaluated in the true complex field environment. Our strategy is based on the observation that the best rootstock in the field is that which combines optimum levels of abiotic tolerance to numerous factors, biotic resistance to numerous diseases, moderate growth traits, and favorable physiological influence on tree nutrition and fruit. This optimum combination is most efficiently identified by direct field trials involving all factors relevant to the production environment, rather than focused testing of individual traits. Concurrent or subsequent testing on those individual factors is most efficient when it can be focused on those few individual new hybrids that clearly exhibit the most outstanding field performance. Information from the focused testing can then be used to define the strengths, weaknesses, and limits of likely production conditions for each of those candidate rootstocks.

## Expanding the Gene Pool by Eliminating Nucellar Embryony as a Required Trait

During the earliest citrus cultivation, trees were primarily grown as seedlings, and nucellar polyembryony was a prerequisite in selection of which citrus types were repeatedly used over time. During the 19th century, the advantages of grafting for maintaining fruit traits, promoting early flowering, and allowing the combination of the best fruit traits with the best root traits became recognized. Using a rootstock (such as sour orange) that propagates easily and uniformly from seed was a big advantage in obtaining a large planting of easily managed productive trees. Consequently, early citrus nursery production quickly focused on rootstock varieties that produced genetically uniform seedlings by nucellar polyembryony. By the time attention was being given to creating new hybrids that might be used as rootstocks, the great convenience of a citrus rootstock that could be propagated uniformly by seed was demanded by citrus nurseries and has heavily focused rootstock breeding efforts for most of the past 100 years. Crosses in which at least one parent did not have and transmit nucellar polyembryony to progeny were avoided for rootstocks, and progeny were not evaluated as potential rootstocks until they had fruited and seedling reproduction could be assessed. Moreover, hybrids that did not themselves exhibit nucellar polyembryony in their seeds were excluded from further testing. Although there are a few examples of citrus used as rootstocks which had less than 50% nucellar seed (Bowman et al., 1995; Rodriguez et al., 2005) and vegetative propagation was possible for most germplasm (Bowman et al., 1997), clones that did not have a high incidence of nucellar seed production were generally not tested as potential rootstocks, and none have become of major commercial importance.

Beginning from previously described methods for stem cutting propagation of citrus (Ferguson et al., 1985), modified methods were developed in the USDA citrus rootstock program to expand both the genetic diversity of hybrid clones that could be used and to increase the percentage of success for the stem cuttings. Initial work resulted in vegetative propagation of numerous new hybrids for field trials, and eventually resulted in the commercial release of three new HLB-tolerant hybrid rootstocks that do not exhibit nucellar polyembryony and cannot be uniformly propagated by seed (Bowman and McCollum, 2015; Barcelos-Bisi et al., 2020). The methodology for citrus stem cuttings evolved through this process and has been described in detail (Bowman and Albrecht, 2017). The new methods are broadly applicable to all citrus types, although frequency of rooting varies by clone. We have found that more than 95% of all clones and hybrids can be successfully propagated by stem cuttings using these methods, allowing it to be employed for replicated field trial testing of nearly any citrus clone as a rootstock. In a recent propagation cycle, more than 5000 single node stem cuttings were made from a diverse collection of 72 citrus cultivars and new hybrids. Three months later, 71 of the 72 clones had produced some growing rooted cuttings, and growing cuttings were obtained from 58% of the cuttings overall. Only 3% of the clones yielded less than 10% healthy plants. Although a high rate of success in stem cutting propagation would be needed for commercial use, even 10% success in rooting is adequate to effectively propagate new rootstock hybrids for field trials. For reference, Swingle, Cleopatra, and sour orange yielded 99%, 56%, and 33% of healthy plants from cuttings, respectively.

**FIGURE 1:**
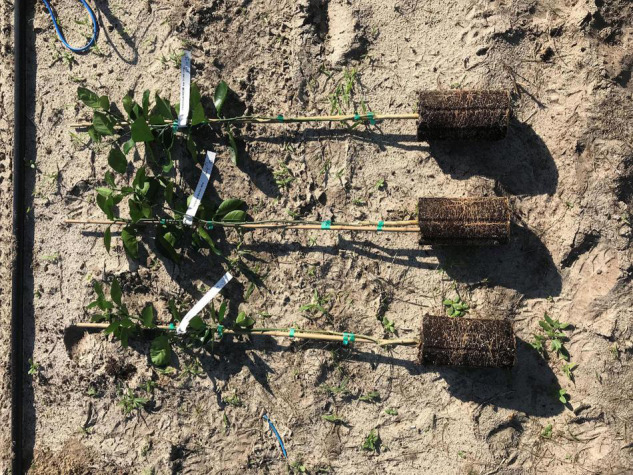
Valencia sweet orange nursery trees at planting time on US-942 rootstock propagated by seed (top), tissue culture (center), and stem cuttings (bottom). Rootstock propagation type appears to have little effect on citrus rootstock performance.

The stem cutting methodology is most suitable for small-scale propagation of a rootstock clone (10–1000 individual rootstock plants), while tissue culture propagation (Carimi and De Pasquale, 2003; Prakash and Sharma, 2018) is more suitable for larger-scale (>5,000 plants) production of a rootstock. Historically, there was concern about whether the characteristics and health of rootstocks propagated by stem cuttings and tissue culture would be as good as that of rootstocks propagated by seed. But rootstocks propagated by all three methods (seed, cuttings, and tissue culture) have been studied intensively, with clear indication that nursery and field performance are primarily determined by the genetic traits of the rootstock cultivar, and only minimally affected by propagation method (Albrecht et al., 2017, 2020; Pokhrel et al., 2021). In one current field trial with Valencia scion and four rootstock clones, a comparison of the rootstocks propagated by nucellar seedlings, stem cuttings, and tissue culture (Figure 1) demonstrated a significant effect of rootstock clone on young tree growth and canopy health, but no significant effect from rootstock propagation type (Table 2). During the 12-month period from July 2019 to June 2020, Florida Department of Agriculture and Consumer Services (FDACS) records indicated that 20% of new propagations (770,000 trees) in Florida used tissue culture propagated rootstock plants, and 6% of new propagations (220,000 trees) used rootstocks propagated by stem cuttings (Rosson, 2020). Although seed propagation is still dominant in commercial use and less expensive than propagating by cuttings or tissue culture, there is a growing broad acceptance of citrus rootstocks propagated by alternative vegetative methods.

**TABLE 2:** Canopy volume, increase in trunk cross sectional area (ΔTCSA), and canopy health rating of ‘Valencia’ trees on different rootstocks propagated by seed (SD), cuttings (CT), and tissue culture (TC) after 18 months in the field.

Factor	Canopy volume (m^3^)	ΔTCSA (mm^2^)	Canopy health rating
**Propagation method**			
SD	0.248	518	4.25
CT	0.236	496	4.23
TC	0.218	444	4.15
*P-*value	0.3022	0.0904	0.8282
**Rootstock cultivar**			
US-1516	0.275 a	517 a	4.45 a
US-812	0.244 a	512 a	4.20 a
US-897	0.131 b	318 b	3.67 b
US-942	0.287 a	599 a	4.53 a
*P-*value	<0.0001	<0.0001	<0.0002
**Propagation method × rootstock**			
*P-*value	0.3084	0.1029	0.8668

*Different letters within columns indicate significant differences according to Tukey’s HSD test at 5% level.*

Some species of citrus (especially those species of hybrid origin, such as *C. sinensis*, *C. aurantium*, and *C. paradisi*) produce predominantly polyembryonic seeds. Other species and selections lack nucellar embryony and produce entirely zygotic seeds, including *C. maxima* (pummelo), *C. medica* L.(citron), some *Citrus* species in the subgroup Papeda, some mandarin species (including *C. clementina* Hort. Ex Tan., *C. tachibana* Tan., some *C. reticulata*), and species in the sexually compatible genus *Microcitrus* (Swingle and Reece, 1967). Evidence has been presented that suggests the simple genetic control of polyembryony in some crosses (Cameron and Soost, 1979), while in other crosses the genetic basis of the trait appears to be more complex (Garcia et al., 1999; Kepiro and Roose, 2010). In breeding practice, hybrid progenies that include even one monoembryonic (zygotic) parent often have few hybrids that possess a level of nucellar polyembryony and apomictic seed that would be acceptable for commercial uniform seed propagation. Although methods are available that allow separation of zygotic from apomictic seedlings in the nursery (Barcelos-Bisi et al., 2020), these are not economically practical at a commercial nursery scale for many types of hybrids. In addition, some hybrids produce few fruit or few seeds per fruit, another way in which outstanding rootstocks may not be suitable for seed propagation.

As described above, in previous citrus rootstock breeding efforts, one of the factors considered essential for a citrus rootstock was the ability to be propagated uniformly by seed (Bitters, 1986), and germplasm that did not transmit nucellar polyembryony to hybrids was rarely used as a parent in rootstock breeding. Under the new SuperSour rootstock breeding strategy, hybridization for new rootstocks is expanded to include parental material that does not exhibit nucellar polyembryony, or does not transmit this trait to progeny. During the early stages of crosses under the new strategy, the gene pool was expanded primarily by including *C. maxima*, *C. ichangensis*, *C. latipes*, mono-embryonic mandarins, and *Microcitrus* spp. among the parental material. Based on our work with vegetative propagation of rootstocks by stem cuttings and tissue culture, any rootstocks with superior field performance can be effectively used on a commercial scale without any need for seed propagation in the commercial nursery.

## Accelerated Field Testing by Propagating Juvenile Hybrids With Stem Cuttings

In addition to the limitation on genetic diversity that resulted from a focus on the need for nucellar seed, this requirement also resulted in a large delay in the initiation of field-testing of new hybrids as rootstocks. In previous rootstock breeding efforts, new hybrid progeny from crosses were planted into the field for “fruiting out” as a first stage in the selection process. It was only after those hybrids produced fruit, that new selections were propagated as seedlings, and entered into specialized testing and replicated field trials (Hutchison, 1976, 1985; Cameron and Soost, 1986). This delay in testing of new rootstock hybrids until seed were produced (and confirmed to be genetically uniform), varied by parentage and conditions, but typically was at least 5 years, and often 10–15 years.

With the SuperSour strategy, young hybrid seedlings are selected during the first 1–2 years in the greenhouse for strong healthy growth, and propagated by stem cuttings for further testing (Bowman and Albrecht, 2017). This eliminates the 5- to 15-year delay to begin testing and replicated field trials with new hybrid rootstocks. Under the new strategy, replicated trees of each rootstock are produced on healthy rooted cuttings, using standard budding methods and a standard sweet orange scion (Valencia 1-14-19 or Hamlin 1-4-1) in a certified greenhouse nursery. The sweet orange trees are subsequently planted into replicated field trials alongside trees on other new hybrid rootstocks and commercial standard rootstocks in one of the three main Florida production regions (East Coast, Central Ridge, Southwest). Propagations of the new SuperSour hybrid clones are also used concurrently to evaluate tolerance to specific diseases and abiotic factors in the field and greenhouse (Bowman and Garnsey, 2001; Albrecht and Bowman, 2004; Graham et al., 2007; Bowman and Albrecht, 2020), and to maintain a clean greenhouse source of vegetative material for continuing propagation and study. Fruiting trees of rootstock selections that demonstrate outstanding field performance may be used as parents in the next generation of rootstock hybrids within the USDA breeding program.

Clean greenhouse source trees of hybrid rootstock selections that demonstrate outstanding field performance are used to submit the clone to the FDACS Citrus Budwood Program^2^ to establish certified clean source material for eventual Florida industry use. Certified clean source material of new hybrid rootstocks that appear to have potential in other production regions are submitted to the California Citrus Clonal Protection Program^3^ and the USDA National Clonal Repository for Citrus, for inclusion in those programs. Propagations of the new SuperSour hybrid clones are also used to plant trees into the field for fruiting out and evaluation of nucellar polyembryony, and the potential for commercial seed propagation. Although the potential for uniform seed propagation is eventually determined for the SuperSour hybrids, it is not a selective factor in determining which hybrids will go into replicated field trials, or a key factor in decisions about which selections may be released for commercial use.

The use of stem cuttings to conduct replicated evaluations of new hybrid rootstocks and establish replicated field trials has already resulted in the successful identification of five new promising rootstocks that were released for commercial use in Florida (Bowman and McCollum, 2015). Subsequent evaluation of seeds from fruiting trees of the five rootstocks revealed that two of them can be uniformly seed propagated, while the other three cannot (Barcelos-Bisi et al., 2020). However, because of the ease of alternative propagation by stem cuttings or tissue culture, uniform seed propagation is not an important factor in evaluating the commercial potential for these five new hybrid rootstocks.

## Other Advantages of Eliminating Focus on Nucellar Seed Propagation

In addition to allowing the expansion of the gene pool for rootstock breeding and the rapid initiation of field trials, eliminating a focus on nucellar seed propagation presents at least two other advantages for the SuperSour strategy. One advantage is that it allows greater focus on other traits of horticultural importance within the rootstock selection process. The previous focus on nucellar polyembryony as a primary selective factor resulted in all other factors (including resistance to biotic and abiotic disease, and fruit productivity) being of secondary importance during the selection process. New hybrids were only considered as potential rootstocks if they possessed the trait of nucellar polyembryony, and consequently many hybrids with potentially very good disease resistance and very high productivity were never considered for further study. By eliminating nucellar embryony as a selective factor, more focus can be given to selection for traits with much greater horticultural importance.

The second advantage is that the recognition of vegetative propagation (cuttings or tissue culture) as an acceptable alternative for citrus rootstocks provides opportunities for much more rapid scale-up of nursery production with the newest outstanding rootstocks. When using seed propagation, there is a delay of several years after seed source trees for a superior rootstock are established and significant quantities of seed are available. We might anticipate that newly planted seed trees for a rootstock will produce no fruit in the first season, 10 fruit per tree after 24 months, and 30 fruit per tree after 36 months. If 250 seed trees were initially planted (about half a hectare) and the rootstock produces 10 seed per fruit, that would equal about 25,000 rootstock seedlings that could be grown after 24 months, and another 75,000 seedlings after 36 months. Estimates of the rate of micropropagation can vary widely, but starting with those same 250 buds to create the seed trees, and using an estimate of citrus tissue culture shoot multiplication at 3.8 × per 4 weeks (Sen and Dhawan, 2010), it would be possible to create more than one million rootstock plants in less than 12 months. Vegetative propagation by tissue culture of new citrus rootstocks allows a much more rapid scale-up of plant production in the nursery than is possible by nucellar seed production.

Taken together, while apomictic seeds of citrus rootstocks are convenient for commercial nursery production, the focus on this trait in commercial rootstock selection has eliminated large segments of the citrus gene pool from use in development of new hybrid rootstocks, greatly delayed the beginning of testing new rootstocks, and diverted focus away from the more important horticultural traits. Eliminating this focus as a selection factor under the SuperSour strategy is a major positive change that will improve the opportunity to identify new rootstocks with superior horticultural traits and significantly accelerate the large-scale commercial use of the newest and best rootstocks.

## Expanded Concurrent Stage 1 Field Testing of New Hybrids

Most documented systematic comparisons of citrus rootstocks in field trials were established as individual trials, and compared an assortment of different existing and new rootstocks in a replicated planting over at least four harvest seasons (Wutscher and Bowman, 1999; Forner-Giner et al., 2003; Louzada et al., 2008; Cantuarias-Avilés et al., 2010; Bowman et al., 2016b; Continella et al., 2018). Typically, trials have contained 4–7 statistical replications of 10–24 different rootstocks, often including existing rootstocks common to the area and selected new or imported rootstocks from other regions. In many situations this was useful and helped define the best among current rootstock options. However, these individual trials with relatively few rootstocks are not suitable for a strategy to compare hundreds of new rootstocks with a limited amount of time and resources. Other trials focused on comparisons of larger numbers of rootstocks were much larger in total area and required much larger commitments of resources to maintain the trials and collect the needed data (Castle et al., 2011; Kunwar et al., 2021).

The new SuperSour strategy employs a series of linked trials to greatly increase the number of rootstocks that can be compared among each other. The trials are linked by having a common scion (sweet orange), a similar experimental design, and several common rootstocks as points of reference, to allow relative comparisons among the rootstocks in all the trials. This provides the opportunity for the effective concurrent testing of hundreds of rootstocks in multiple trials, and generates performance data that can be systematically compared between the trials. Between 2014 and 2021, there were 15 of these linked SuperSour trials with sweet orange scion planted in Florida, containing 350 new hybrid rootstocks along with four standard rootstocks for cross-comparison in all the trials. Reference of results in each trial to the standard rootstocks will allow multi-trial comparisons of rootstocks and improve selection of new rootstocks that are best overall, not just in individual trials. The average number of new hybrid rootstocks in each trial is 50, average rootstock replications per trial is nine, and most of the 350 hybrid rootstocks are included in more than one trial.

**FIGURE 2:**
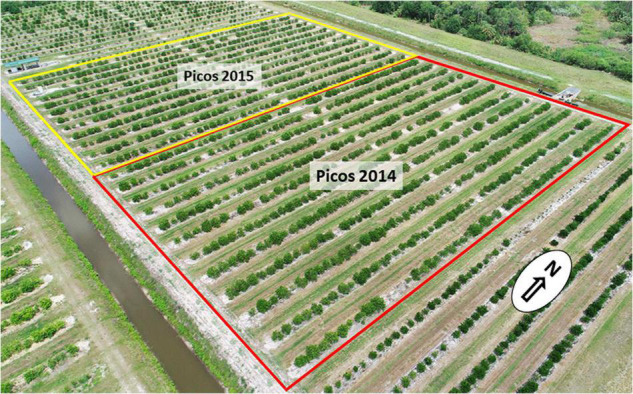
Aerial image in May 2021 of the two rootstock trials with Valencia sweet orange scion, planted with 1287 trees (Photo by R. Niedz).

Two of the 15 trials have been chosen to illustrate the strategy and results obtained from the trials. The two representative trials are in adjacent sections at the USDA Picos Farm in Fort Pierce, Florida, which is on the East Coast of Florida where poorly drained sandy soils are typical (Mylavarapu et al., 2019). One of the trials was planted in October 2014 and the other in October 2015, and named ‘Picos 2014’ and ‘Picos 2015,’ respectively (Figure 2). Each trial was planted on 12 rows, with about 640 trees per trial. The trials are planted on leveled double row beds, with good drainage and microsprinkler irrigation, as is standard practice for this area. Rootstocks for the trials were propagated by stem cuttings (Bowman and Albrecht, 2017) from the hybrid seedlings, and trees produced by budding with certified Valencia 1-14-19 sweet orange budwood using standard methods in the certified greenhouse citrus nursery at the US Horticultural Research Laboratory in Ft. Pierce, FL, United States. Management of trees in the two blocks employed common fertilization, weed, and pest control practices for citrus in this area. As is normal for field plantings in Florida citrus production areas where CLas is endemic (Graham et al., 2020), PCR surveys of trees in the block indicated that within the first 3–4 years after planting, 100% of trees in the trials were infected with CLas. A mild strain of CTV is also endemic in the area of these trials, although usually there are no negative effects on sweet orange trees from infection.

Detailed annual performance information on each replication is collected from the SuperSour field trials, including measurements of tree survival and size, canopy health, quantity of fruit produced, and fruit quality (brix, acid, percent juice, color, and fruit size). Performance traits of the greatest interest in citrus rootstock trials, and approach to analysis of results can vary by situation and focus. However, generally the traits tree health, fruit yield, yield efficiency, pounds solids per box (PSB), juice color, and brix:acid (BA; soluble solids:titratable acidity) ratio are of great importance in assessing the performance of rootstocks in Florida field trials with sweet orange scion being grown for juice production. Within both trials, significant differences were observed among the rootstocks for all six of these traits (see Supplementary Tables 1, 2).

**FIGURE 3:**
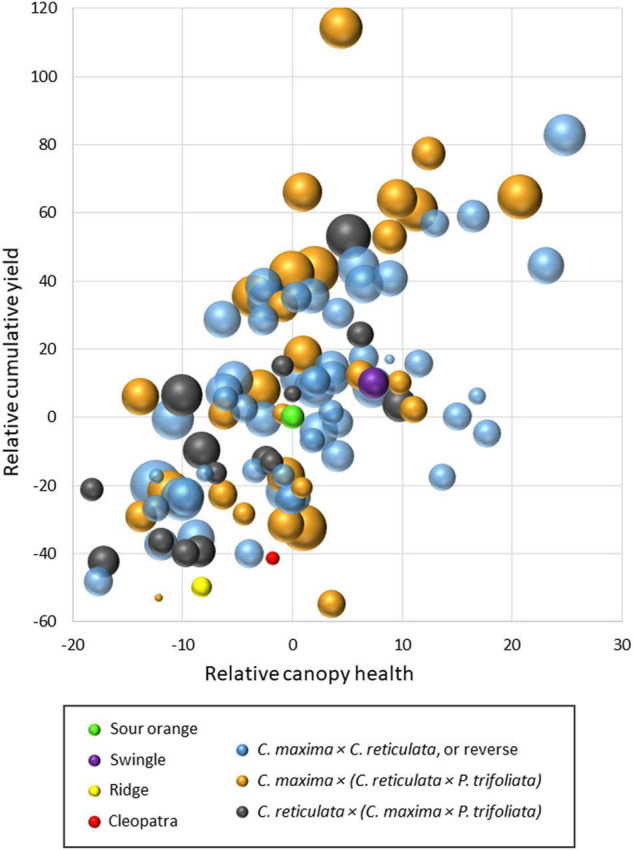
Comparison of cumulative yield, canopy health, and yield efficiency (bubble size) for 97 new SuperSour hybrid rootstocks relative to standard sour orange rootstock and three other commercial rootstocks in two trials with Valencia scion, planted in 2014 and 2015.

To illustrate overall tree performance in the two trials, results were transformed to make relative comparisons with the standard rootstock sour orange within each trial for the traits: (1) cumulative fruit yield (over seasons 4–6), (2) canopy health (subjective score of canopy density and leaf color), and (3) yield efficiency (ratio of fruit yield:canopy size). Results are presented in a bubble chart (Figure 3), with all values transformed to make sour orange values zero for cumulative yield and canopy health. Values for yield efficiency were transformed so that sour orange yield efficiency produced a medium-size bubble). In the chart, the mean value for each rootstock relative to sour orange was represented by a single bubble, and results are combined from the two trials. Rootstocks which induced relatively higher or lower values for cumulative yield, canopy health, and yield efficiency than sour orange are represented according to those differences by position on the x-y axis and by bubble size. In total, Figure 3 compares values for sour orange rootstock with three other standard rootstocks (Swingle citrumelo, Cleopatra [*C. reticulata*], and Ridge [*C. sinensis*]) and 97 SuperSour hybrid rootstocks. Included in this summary are data from 59 hybrids plus the four standard rootstocks in Picos 2014, and 44 hybrids plus the four standard rootstocks in Picos 2015 (six of the hybrids were in both trials). In these two trials, three different types of parental combinations were included among the new SuperSour hybrids being compared, and are indicated by bubble color on the chart:

• *C. maxima* × *C. reticulata*, or the reverse
• *C. maxima* × (*C. reticulata* × *P. trifoliata*)
• *C. reticulata* × (*C. maxima* × *P. trifoliata*).

More than half of the progeny from all three parental combinations were superior to sour orange rootstock for cumulative yield, canopy health, and yield efficiency. Among the other three standard rootstocks in the trials, Swingle was slightly better than sour orange for the three traits, while the other two (Cleopatra and Ridge) were worse. This matches well with commercial use, as Swingle and sour orange are among the five most used rootstocks in Florida, while Cleopatra and Ridge have relatively little commercial use. Some of the new SuperSour hybrids demonstrated very large advantages in cumulative yield and yield efficiency as compared with the standard rootstocks. This suggests good potential to identify superior new commercial rootstocks among these progeny types. The comparison of performance from progenies of the three parental combination types may suggest relative differences in potential from different types of crosses, which will be the subject of further study.

**FIGURE 4:**
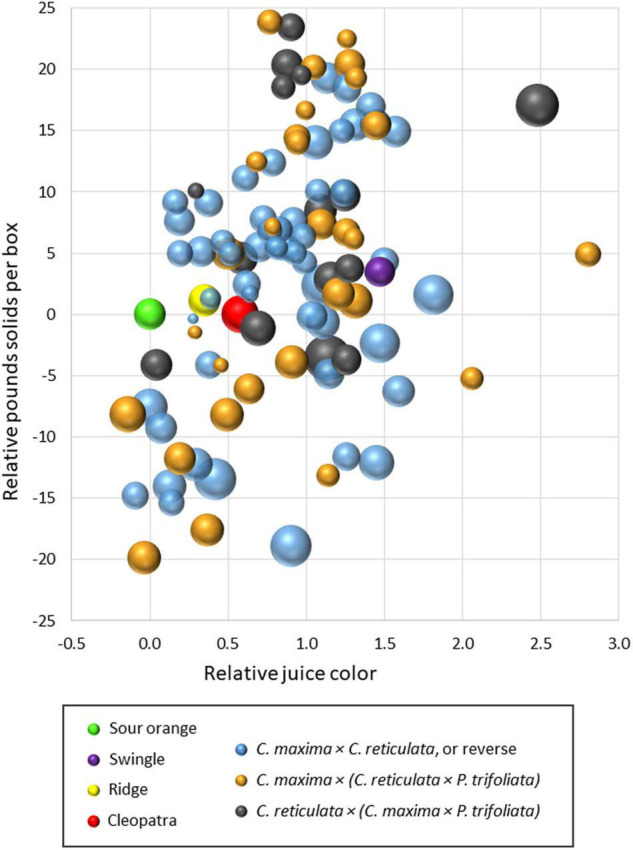
Comparison of fruit pounds solids per box, juice color, and brix/acid ratio (bubble size) for 97 new SuperSour hybrid rootstocks relative to standard sour orange rootstock and three other commercial rootstocks in two trials with Valencia scion, planted in 2014 and 2015.

A similar comparison was made in these same two trials on the influence of rootstock on fruit quality of the Valencia sweet orange scion during the 2019–21 seasons. Results within each trial were transformed to make relative comparisons with the standard rootstock sour orange for the traits: (1) PSB, (2) juice color (CN color scale), and (3) juice BA ratio. Results are presented in a bubble chart (Figure 4), with all values transformed to make sour orange values zero for PSB and juice color. Values for BA ratio were transformed so that sour orange BA ratio produced a medium-size bubble. As in Figure 3, the mean value for each rootstock relative to sour orange was represented by a single bubble, and results are combined from the two trials. Figure 4 compares fruit quality values over three harvest seasons for sour orange rootstock with three other standard rootstocks (Swingle, Cleopatra, and Ridge) and 97 SuperSour hybrid rootstocks. More than half of the SuperSour hybrids induced a higher PSB than sour orange, and the PSB value for the other standard rootstocks were similar to that for sour orange. For influence on Valencia juice color, sour orange was among the worst of the rootstocks, and nearly all of the SuperSour hybrids (and the other three standard rootstocks) exhibited a superior influence on juice color. Most notably, Swingle was among the rootstocks that had the strongest positive influence on juice color. Although some of the hybrids were similar, sour orange was among the best of the rootstocks for influence on BA ratio, suggesting that this trait may be the greatest challenge to achieve for a superior new rootstock.

It appears from these results that many of the hybrid rootstocks among these three different types of SuperSour parental combinations may be superior in many respects to the common commercially used rootstocks in Florida, like sour orange and Swingle. Relative comparisons among these two trials suggest that results from the larger group of 15 trials can be cross-compared and used effectively to identify those rootstocks with superior positive attributes for potential commercial release. Comparisons of rootstock performance under different conditions at different locations is included as a part of the SuperSour strategy, but is more complex, and is not included in this illustration. Individual relative deficiencies of particular hybrids that are otherwise outstanding can be clearly identified and evaluated for relative significance in commercial use. In some cases, relative weaknesses (for example, relatively low color score) might be judged of minor significance for a rootstock that otherwise induced high yield, high yield efficiency, and high PSB.

## Identify Superior Parental Combinations and Map Rootstock Traits

One large long-term benefit of the new SuperSour strategy, and the concurrent testing of hundreds of related hybrid rootstocks from several different parental combinations, is the opportunity to identify those parental combinations that have the highest likelihood to produce progeny with particularly important rootstock traits or combinations of important rootstock traits. Although previous field trials and resultant datasets related to citrus rootstock traits were inadequate to evaluate the combining ability of potential citrus parents (the ability to transmit positive rootstock attributes to progeny), accurate estimates of general and specific combining ability are widely considered a critical tool in plant breeding (Allard, 1960; Oakey et al., 2006; Fasahat et al., 2016). Information about the combining ability of numerous specific citrus parents or types of parents would be an immensely valuable tool to help guide the next generations of citrus rootstock breeding. Among the 97 hybrid progeny in the two-trial illustration (Picos 2014 and Picos 2015), there were large differences in mean values for the three progeny groups that were compared. For example, the population of *C. maxima* × (*C. reticulata* × *P. trifoliata*) hybrid progeny tested in those two trials had a mean value for cumulative yield of plus 13.8 units, while the mean cumulative yield value from the progeny of the cross *C. reticulata* × (*C. maxima* × *P. trifoliata*) had a mean value of minus 8.1 units. While this is only a preliminary comparison from a partial dataset, it suggests that one parental combination is likely to create hybrids with higher yield than the other parental combination. The progeny groups developed under the SuperSour strategy, with corresponding field trials, will generate the needed datasets to identify optimum parental combinations and utilize estimates of combining ability as a tool to help guide next-generation citrus rootstock breeding.

Related to knowledge about combining ability, the extensive datasets for rootstock performance information of a large group of citrus hybrids can also be used to effectively map genes associated with important rootstock traits on the citrus genome. Molecular markers, association mapping, and quantitative trait locus mapping are common tools in plant breeding of annual crops, made practical by the use of test crosses and very short generation times (Xu and Crouch, 2008; Ibrahim et al., 2020). Recent advances in data analysis demonstrate that similar mapping of genes can be accomplished from unbalanced and diverse multi-environment field trial data (Rife et al., 2018; MacQueen et al., 2020), such as the data from the SuperSour strategy with 15 concurrent sweet orange rootstock trials and 350 hybrid rootstocks. We plan to use this multi-trial, multi-year data to map rootstock influence on tree survival, tree size, tree canopy health, disease tolerance, fruit productivity, and a range of important fruit quality traits, and then create easily applicable molecular markers to aid selection of future superior hybrid rootstocks in the breeding program. Selection using molecular markers associated with multiple important rootstock traits (such as survival and productivity) will allow field trials to focus on a pre-selected group of hybrids with the best genetic potential to be outstanding.

## A Continuum to Commercial Use

The SuperSour strategy makes use of more diverse and elite germplasm, eliminates preselection for apomictic seed production, integrates rapid vegetative propagation for early testing of new hybrids, and evaluates very large numbers of new hybrid rootstocks in multiple concurrent trials to allow relatively rapid comparisons of large numbers of new hybrids for good rootstock traits. Preliminary evaluation of results from two of these trials indicates that results from the individual trials and cross-comparisons between trials can be used to effectively identify positive and negative attributes for each hybrid clone and to select the most superior rootstocks for commercial release. Results from the trials will be used to evaluate the usefulness of several possible selection indices, which may more clearly identify rootstocks with the best combination of important traits. This same information can be used for development of genetic maps and molecular markers to speed the next cycle of rootstock development.

While the SuperSour strategy empowers more rapid development of new rootstocks with more diverse genetics, it is not the end of the rootstock development process. Knowledge about rootstock tolerance or resistance to a particular abiotic stress (flooding, drought, salinity, or cold) and biotic stress (nematodes, CTV, *Phytophthora*, or CLas) is of great value, as it helps to define critical limitations to conditions under which the new rootstock should be used. Within the USDA program, this focused testing is conducted on the most outstanding of the candidates as they reach the end of SuperSour strategy field trials. Rootstocks with individual faults may be of commercial value, as long as the limit of that fault is understood. Similarly, the graft compatibility of the new rootstocks with the broad range of commercially important scions needs to be assessed, to avoid unanticipated graft-incompatibility in the commercial nursery or in the field. Within the SuperSour strategy, focused trials containing the new rootstocks grafted with the major scion cultivar types are conducted to complement the field trials with sweet orange scion. It may also be noted that superior new rootstock cultivars that cannot be uniformly propagated by seed will be much slower to be used in new citrus growing regions because of the extensive inter-region quarantine regulations that limit movement of vegetative plant material (Lavagi-Craddock et al., 2021).

The SuperSour strategy allows the development of new rootstocks in as little as 8 years from cross to release. However, additional time is needed to fully evaluate nursery traits, tolerance to abiotic and biotic stresses, and compatibility with a range of different scions. Depending on the production situation, commercial use with sweet orange in the tested environments would be appropriate immediately, while commercial use with other scions or in other situations may await further trials and testing. The intention of the SuperSour strategy is to create a cycle that repeatedly creates promising new hybrids from elite and exotic germplasm, establishes new trials, conducts specialized testing, and then uses data about those hybrids in the trials and testing to identify superior rootstocks for commercial release, and guide the next cycle of rootstock breeding.

## Author Contributions

KB conceptualized the work, wrote the first draft of the manuscript, and critically edited the manuscript for publication. GM and UA conducted important portions of the work described in the manuscript and edited and added to the manuscript. All authors contributed to the article and approved the submitted version.

## Conflict of Interest

The authors declare that the research was conducted in the absence of any commercial or financial relationships that could be construed as a potential conflict of interest.

## Publisher’s Note

All claims expressed in this article are solely those of the authors and do not necessarily represent those of their affiliated organizations, or those of the publisher, the editors and the reviewers. Any product that may be evaluated in this article, or claim that may be made by its manufacturer, is not guaranteed or endorsed by the publisher.

## Footnotes

1
https://programs.ifas.ufl.edu/plant-breeding/citrus/

2
https://www.fdacs.gov/

3
https://ccpp.ucr.edu/
