# Supplementary material for: SuperSour: A New Strategy for Breeding Superior Citrus Rootstocks
Source: Front Plant Sci. 2021 Nov 4;12:741009. doi: 10.3389/fpls.2021.741009 (PMC8600239; doi:10.3389/fpls.2021.741009)
Supplement: Supplementary file 1 [file Data_Sheet_1.PDF]

Supplementary Table 1. Summary of ANOVA results from the Picos 2014 trial.

Cumulative yield (kg)

	Df	SS	MS	F value	p>F
Treatment	62	26333.81	424.7388	4.7731	<0.001
Residuals	481	42802.34	88.9861		

Sour orange mean cumulative yield ± SE = 20.72 ± 2.36

Canopy health (scale of 1-5)

	Df	SS	MS	F value	p>F
Treatment	62	60.4771	0.9754	2.7636	<0.001
Residuals	481	169.7760	0.3530		

Sour orange mean canopy health ± SE = 3.52 ± 0.15

Yield efficiency (kg/m^3)

	Df	SS	MS	F value	p>F
Treatment	62	679.7622	10.9639	1.6546	0.0021
Residuals	481	3187.2566	6.6263		

Sour orange mean yield efficiency ± SE = 2.23 ± 0.64

Juice color (CN scale)

	Df	SS	MS	F value	p>F
Treatment	62	11.5746	0.1867	1.6799	0.0016
Residuals	471	52.3423	0.1111		

Sour orange mean juice color ± SE = 36.50 ± 0.08

Pounds solids per box (Total soluble solids per 90 pounds of fruit)

	Df	SS	MS	F value	p>F
Treatment	62	26.2257	0.4230	4.3209	<0.001
Residuals	471	46.1085	0.0979		

Sour orange mean lbs solids per box ± SE = 3.41 ± 0.08

Brix/acid ratio

	Df	SS	MS	F value	p>F
Treatment	62	145.7059	2.3501	1.3472	0.0478
Residuals	471	821.6199	1.7444		

Sour orange mean brix/acid ratio ± SE = 11.47 ± 0.33

Supplementary Table 2. Summary of ANOVA results from the Picos 2015 trial.

Cumulative yield (kg)

	Df	SS	MS	F value	p>F
Treatment	47	13527.05	287.8096	2.85	<0.001
Residuals	462	46655.37	100.9857		

Sour orange mean cumulative yield ± SE = 15.61 ± 2.90

Canopy health (scale of 1-5)

	Df	SS	MS	F value	p>F
Treatment	47	31.3254	0.6665	2.4197	<0.001
Residuals	462	127.2561	0.2754		

Sour orange mean canopy health ± SE = 3.14 ± 0.15

Yield efficiency (kg/m^3)

	Df	SS	MS	F value	p>F
Treatment	47	943.2482	20.0691	3.2705	<0.001
Residuals	462	2835.0061	6.1364		

Sour orange mean yield efficiency ± SE = 3.82 ± 0.72

Juice color (CN scale)

	Df	SS	MS	F value	p>F
Treatment	47	20.5919	0.4381	3.2954	<0.001
Residuals	313	41.6140	0.1330		

Sour orange mean juice color ± SE = 36.71 ± 0.14

Pounds solids per box (Total soluble solids per 90 pounds of fruit)

	Df	SS	MS	F value	p>F
Treatment	47	28.1898	0.5998	4.4261	<0.001
Residuals	313	42.4146	0.1355		

Sour orange mean lbs solids per box ± SE = 3.92 ± 0.14

Brix/Acid Ratio

	Df	SS	MS	F value	p>F
Treatment	47	242.2898	5.1551	1.5568	0.0153
Residuals	313	1036.4753	3.3114		

Sour orange mean brix/acid ratio ± SE = 11.46 ± 0.69
